# Interleaflet Coupling of Lipid Nanodomains – Insights From *in vitro* Systems

**DOI:** 10.3389/fcell.2020.00284

**Published:** 2020-04-28

**Authors:** Maria J. Sarmento, Martin Hof, Radek Šachl

**Affiliations:** J. Heyrovský Institute of Physical Chemistry of the Czech Academy of Sciences, Dolejškova, Prague, Czechia

**Keywords:** domain registration, interleaflet coupling, membrane asymmetry, nanodomains, plasma membranes, phase separation, biomembranes

## Abstract

The plasma membrane is a complex system, consisting of two layers of lipids and proteins compartmentalized into small structures called nanodomains. Despite the asymmetric composition of both leaflets, coupling between the layers is surprisingly strong. This can be evidenced, for example, by recent experimental studies performed on phospholipid giant unilamellar vesicles showing that nanodomains formed in the outer layer are perfectly registered with those in the inner leaflet. Similarly, microscopic phase separation in one leaflet can induce phase separation in the opposing leaflet that would otherwise be homogeneous. In this review, we summarize the current theoretical and experimental knowledge that led to the current view that domains are – irrespective of their size – commonly registered across the bilayer. Mechanisms inducing registration of nanodomains suggested by theory and calculations are discussed. Furthermore, domain coupling is evidenced by experimental studies based on the sparse number of methods that can resolve registered from independent nanodomains. Finally, implications that those findings using model membrane studies might have for cellular membranes are discussed.

## Introduction

Plasma membranes are composed of two layers of lipids and proteins in close contact with each other. They are in fact so close that the individual layers cannot be viewed as two independent units. In literature, this physical phenomenon is known as interleaflet coupling (Devaux and Morris, 2004; Collins, 2008; Kiessling et al., 2009; May, 2009; Fujimoto and Parmryd, 2017) and has in the past been evidenced through experiments showing that the physical properties of one layer can modulate the physical state of the opposing leaflet (Chiantia and London, 2012; Heberle et al., 2016).

Importantly, biological membranes are not homogeneous. They contain small heterogeneities, also known as lipid nanodomains (Eggeling et al., 2009; Owen et al., 2012), with different physico-chemical properties in respect to the surrounding bulk membrane (Cebecauer et al., 2018). Although lipid nanodomains of similar size have even been detected in synthetic model membranes consisting of only two different types of lipids (Koukalová et al., 2017), they are still difficult to detect and characterize due to their small size (<200 nm) (Cebecauer et al., 2018). Consequently, liquid-ordered (L_*o*_; see Box 1) microdomains (>200 nm) have been extensively used as suitable models for plasma membrane nanodomains, despite their significantly larger size, less dynamic behavior, and increased membrane order (Dietrich et al., 2001; Mouritsen and Bagatolli, 2015; Cebecauer et al., 2018). Interestingly, microdomains formed in one leaflet of free-standing vesicles have always been found in perfect alignment with the microdomains formed in the opposing layer, implying the presence of interleaflet coupling (Garg et al., 2007; Kiessling et al., 2009; Blosser et al., 2015). Furthermore, shear stress experiments performed on supported phospholipid bilayers (SPBs) show that the pressure required to deregister (de-couple) microdomains increases with the decrease in domain size (Blosser et al., 2015). This observation suggests that biologically more relevant nanodomains are also perfectly registered (Garg et al., 2007; Kiessling et al., 2009; Blosser et al., 2015). As shown below in this review, recent Förster resonance energy transfer analyzed by Monte Carlo simulations (MC-FRET) experiments confirm this hypothesis (Vinklárek et al., 2019).

**BOX 1**

Lateral segregation of lipids resulting in membrane heterogeneity.

Depending on the temperature and lipid composition, a lipid bilayer may be present in several phases. *Liquid-disordered phase*, L_*d*_, usually encountered above the melting temperature (*T*_*m*_) of the bilayer is characterized by high lipid mobility with maximal rotational and translational freedom, low lipid packing and disordered acyl chains. By lowering the temperature below the *T*_*m*_, the bilayer freezes into a *solid (gel) phase*, *S*, for which low lipid mobility, high lipid packing and membrane order are typical. In some conditions, usually in bilayers containing cholesterol, *liquid-ordered phase*, L_*o*_, may be formed. With regard to the mobility of individual lipids, this phase rather resembles the L_*d*_ phase, with only slightly reduced diffusion of lipids in comparison to the actual L_*d*_. In contrast, membrane order and lipid packing are more similar to the *S* phase.

Due to limited miscibility of some lipids, a bilayer can separate into several co-existing phases, resulting in membrane heterogeneity. Depending on the size of these heterogeneities, they are termed as *micro- or nanodomains*. In this work and in analogy to (Cebecauer et al., 2018) we define nanodomain as “any compartmentalization within a lipid membrane that has an estimated ‘diameter equivalent’ within the range of 4–200 nm.” Since the nanodomain of 4 nm diameter contains only 100 lipids, it cannot be viewed as a single phase, i.e., a region of space throughout which all physical properties of a material are essentially uniform.

Additionally, plasma membranes are asymmetric (Devaux and Morris, 2004), with most glycosphingolipids (GSL), sphingomyelins (Sph), and phosphatidylcholines (PC) being found in the outer leaflet, whereas phosphatidylinositol phosphates (PIPs), phosphatidylserines (PS), and phosphatidylethanolamines (PE) are mainly localized in the inner leaflet (Bretscher, 1972; Devaux, 1991; Harayama and Riezman, 2018). Although this membrane asymmetry had been recognized a long time ago, asymmetric model membranes only began to be used recently. This is probably due to limitations on the preparation methods and the limited stability of the induced asymmetry. Nevertheless, experiments performed on these systems have clearly shown that the presence of domains in one layer is capable of inducing the formation of registered domains in the other leaflet (Collins and Keller, 2008; Wan et al., 2008; Kiessling et al., 2009; Wang and London, 2018) and that the final strength of interleaflet coupling depends on the actual conditions of the membrane. Not surprisingly, coupling is often mentioned in connection with signal transduction across the plasma membrane (Iwabuchi et al., 2010; Wernick et al., 2010; Bergan et al., 2012; Skotland and Sandvig, 2019), which can be triggered by clustering of receptors (i.e., formation of membrane nanodomains) in the outer leaflet and transfer of information to the inner leaflet (Klokk et al., 2016).

Characterization of coupling in simpler model systems has proved to be a convenient tool to understand the underlying mechanisms that ultimately lead to domain registration. Keeping this in mind, in this review we first discuss the mechanisms suggested to cause interleaflet coupling, potentially resulting in domain registration. In the following section, we have summarized the experimental evidence that contributed to the current understanding that the level of interleaflet coupling is neither negligible nor strong. It is instead moderate yet enough to universally register lipid domains irrespective of their size. Since the characterization of nanodomains’ interleaflet organization requires the use of sophisticated biophysical approaches and up-to-date techniques, we have paid significant attention to the principles based on which the most important techniques can detect domain registration. At the end of the review, we discuss the implications that the discoveries on model systems might have for our overall understanding of the organization and function of the plasma membrane in living cells. Moreover, and despite the sparse evidence, we attempt at discussing how proteins might contribute to lipid nanodomain registration and how these protein–lipid interactions can ultimately be of great significance for signal transduction across different biological membranes.

## Theoretical Framework: Mechanisms Leading to Nanodomain Registration

### Line Tension

**FIGURE 1:**
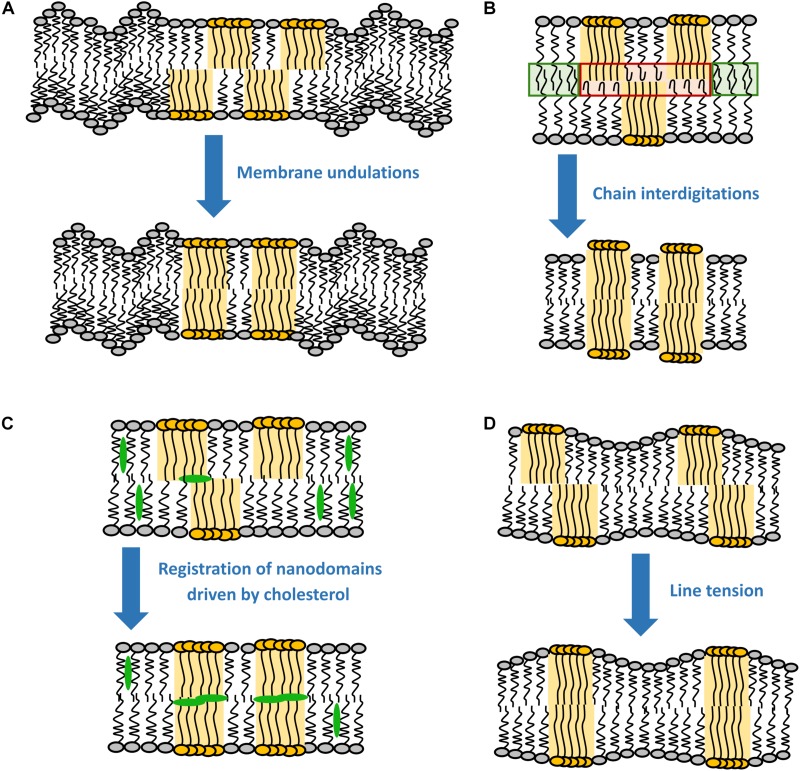
Most probable driving forces for the registration of nanodomains. **(A)** Membrane undulations, **(B)** chain interdigitations, **(C)** registration of nanodomains by cholesterol, and **(D)** line tension.

It has been shown by X-ray diffraction that L_*o*_ domains are thicker than the surrounding liquid-disordered (L_*d*_) phase (see Box 1) (Gandhavadi et al., 2002), meaning that in principle there is a hydrophobic mismatch between the acyl chains of different phases, with the ones in the L_*o*_ phase being more exposed to the hydrophilic environment. This gives rise to line tension, which can be understood as energy stored into a unit length of the domain boundary. For instance, for a 0.5 nm difference in the thickness of the two phases, the line tension would theoretically rise to about 6 k_*B*_T nm^–1^ (Tien, 1974; Galimzyanov et al., 2015). However, such high value has never been observed experimentally, since lipid re-distribution assures a smooth transition between phases and the consequent decrease in the line tension (panel D in Figure 1) (Baumgart et al., 2003; Esposito et al., 2007; Akimov et al., 2009).

**FIGURE 2:**
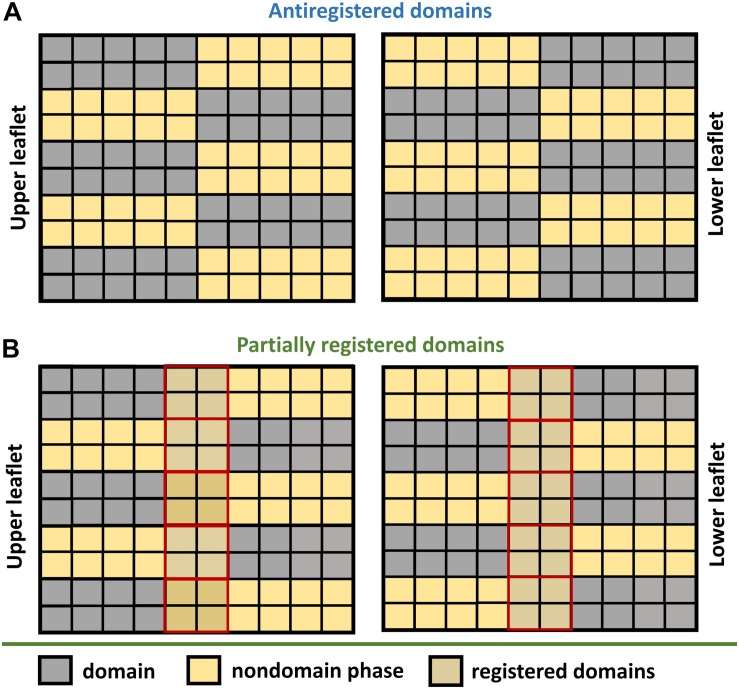
Domain anti-registration versus partial registration. **(A)** Domains are classified as antiregistered when the domains localized in one leaflet (gray squares) are aligned with the nondomain region in the other leaflet (yellow squares). Such an arrangement is possible only if the domain and nondomain subregions occupy an equal area of the bilayer. **(B)** Higher amount of one of the regions inevitably leads to transversal overlap between the domain and nondomain subregions (yellow-gray squares surrounded by solid red).

As shown by Galimzyanov et al. (2015), line tension alone is sufficient to drive registration of nanodomains. However, the efficiency of this process decreases with the increase in nanodomain size (Galimzyanov et al., 2017). In fact, minimal line tension is reached when nanodomains get into registration with a slight shift (∼4 nm) relative to each other (Galimzyanov et al., 2015). This effect is universal, since it does not require any special lipid component in the membrane. Theoretically, the line tension disappears completely if nanodomains get into antiregistration. However, the amounts of L_*o*_ and L_*d*_ phases would need to match perfectly (Figure 2). Naturally, this condition is hardly achievable for an actual membrane, which explains why antiregistration has so far only been observed *in silico* (Perlmutter and Sachs, 2011; Galimzyanov et al., 2015; Williamson and Olmsted, 2015b).

Interestingly, surface tension, which can be induced for instance by bending or swelling of the lipid bilayer, cannot drive domain registration (Akimov et al., 2009; Galimzyanov et al., 2015). In fact, lipid domain registration occurs rather independently of the surface tension applied onto the membrane. Nevertheless, it influences the energy balance of the whole process (Frolov et al., 2006; Akimov et al., 2007; García-Sáez et al., 2007). Specifically, increasing surface tension leads to greater energy storage at the domain boundaries, which in turn favors the coalescence of domains and the subsequent increase of the domain size (Ayuyan and Cohen, 2008; Akimov et al., 2009).

### Membrane Undulations

It follows from theoretical considerations based on continuum theory that the line tension alone is not enough to register nanodomains larger than 38 nm in radius (Galimzyanov et al., 2017). Galimzyanov et al. (2017) identified thermal membrane undulations as an additional energy source contributing to robust coupling of nanodomains for a broad spectrum of nanodomain sizes (panel A in Figure 1). It is known that L_*o*_ domains have approximately 2–3 times higher bending rigidity than the surrounding L_*d*_ phase (Khelashvili et al., 2013; Kollmitzer et al., 2015). Because such stiff parts of both leaflets cannot undulate with the same efficiency as the remaining L_*d*_ phase, membrane undulations drive the stiff regions into the same bilayer locations, causing registration of domains. According to recent experimental work on SPBs, the resulting energy gain is about 0.016 k_*B*_T nm^–2^ (Blosser et al., 2015). Since the gain in the coupling energy increases with the increase in the nanodomain area, undulations primarily act on the registration of larger domains, although they are still able to co-localize nanodomains as small as 10 nm in radius (Haataja, 2017). This behavior is thus utterly the opposite to the line tension, which mainly drives registration of smaller domains (see section “Line Tension”). Interestingly, the efficiency of the undulations varies significantly with the wavelength of the fluctuations, the most efficient being in the ultraviolet region and then rapidly declining with the increase in the wavelength (Galimzyanov et al., 2017).

Undulations represent a robust mechanism of domain registration, being resistant to external influences. For instance, a surface tension as high as 10 nN m^–1^, which is able to rupture a lipid bilayer (Evans et al., 2003), has only a modest effect on the coupling energy. This is mainly because the tension is only able to supress fluctuations with longer wavelengths, which do not contribute to domain registration (Galimzyanov et al., 2017). Similarly, undulations are only slightly affected by a solid membrane support, which is known to hamper collective motion of lipids in both leaflets and slow down motion of individual lipids (Sonnleitner et al., 1999; Przybylo et al., 2006; Garg et al., 2007). Although the theoretical framework for membrane undulations predicts the membrane support to not influence the coupling energy that governs domain registration (Galimzyanov et al., 2017), recent atomic force microscopy (AFM) experiments performed on various membrane supports indicate that it may significantly affect interleaflet coupling of lipid domains (see section “Imaging”).

### Chain Interdigitation

Even though acyl chain interdigitation is often suggested in literature as a plausible mechanism for interleaflet coupling, its importance has not yet been sufficiently confirmed (May, 2009; Nickels et al., 2015b; Fujimoto and Parmryd, 2017; Skotland and Sandvig, 2019) (panel B in Figure 1). In principle, interdigitation is expected to fuel registration of nanodomains because the ability of acyl chains to penetrate (interdigitate) into the opposing leaflet is considerably better if the L_*d*_ phase faces a similar L_*d*_ environment in the opposing leaflet. Interdigitation will be hampered if the disordered phase faces an ordered one, in which the acyl chains cannot penetrate. Therefore, chain interdigitation should be thermodynamically favorable due to the overall entropy increase of the acyl chains. Interestingly, the energy of the interaction between leaflets (0.1–10 k_*B*_T nm^–2^) is similar to the energy that would have to be paid in order to prevent chain interdigitation (Szleifer et al., 1990; Collins, 2008; May, 2009).

In practice, a couple of experiments support the hypothesis that chain interdigitation is important, although not required, for interleaflet coupling. It has been shown by fluorescence correlation spectroscopy (FCS) that the presence of long chain Sph (C24:0) in the outer membrane leaflet slows down lipid diffusion within the inner leaflet composed of 1,2-dioleoyl-*sn*-glycero-3-phosphocholine (DOPC) (Chiantia and London, 2012). Similarly, molecular dynamics (MD) simulations indicate that long chain Sph can penetrate deeply into the opposing leaflet, strongly interacting with neighboring lipids (Róg et al., 2016). Recently, it has also been proposed that chain interdigitation might even have biological significance. Skotland and Sandvik (2019) suggest in their perspective article that toxin-induced clustering of long chain Sph in the outer leaflet could be used to transmit the signal into the inner leaflet, through interdigitation of Sph molecules and their consequent coupling to the lipids with short tails from the inner leaflet. From MD simulations, the strongest of these interactions was observed to be between Sph (18:0/24:0) and PS (16:0/18:1), a well-known signaling lipid (Llorente et al., 2013).

Despite these interesting findings that rather support interdigitation as one of the main coupling mechanisms, there are experiments questioning this concept. For instance, Horner et al. (2013) measured the intermonolayer viscosity of fluorescent probes with both short and long acyl chains and found out that the viscosity was independent of the acyl chain length. This result would in principle rule out interdigitation as one of the main coupling mechanisms. Further supporting this hypothesis, Chiantia and London (2012) have demonstrated by FCS that interleaflet coupling of diffusion does not necessarily require lipids with long acyl chains, but rather occurs in the presence of lipids containing one saturated acyl chain [such as 1-palmitoyl-2-oleoyl-*sn*-glycero-3-phosphocholine (POPC), which is abundant in cellular membranes]. For example, substitution of DOPC by 1-oleoyl-2-myristoyl-*sn*-glycero-3-phosphocholine (OMPC) led to a significant increase in the coupling of diffusion across the bilayer (Chiantia and London, 2012). This suggests that, instead of chain interdigitation, the physical proximity and possible interaction between acyl chains of complementary leaflets at the bilayer midplane can play a more general role, in which strong interdigitation would be the extreme case. In this way, stretching of a saturated acyl chain of, e.g., POPC would also allow a more effective interaction with the slow moving Sph in the outer leaflet (when compared with DOPC), reducing its diffusion and ultimately resulting in interleaflet coupling.

Additional insight into the whole problematics has been provided by MD simulations. First, the simulations do not support complementarity between short chains in one layer and long chains in the opposite layer as the source of interleaflet coupling (Capponi et al., 2016). Second, it has been shown that the acyl chains can move very fast and exhibit a disordered character (Lu et al., 1995). Particularly the oleoyl chains of DOPC can bend back to the interface, thereby reducing the electron density close to the membrane midplane (Chiu et al., 1999). A higher density of terminal methyl groups was found for POPC in comparison to DOPC lipids (Chiu et al., 1999). Moreover, the determined average distance between terminal segments of both leaflets is significantly smaller for bilayers with one saturated chain than for a DOPC bilayer (Chiantia and London, 2012). Nuclear magnetic resonance (NMR) experiments have confirmed that the methylene groups do penetrate into the opposing leaflet and that the chain mobility is only partially affected by the opposing leaflet (Xu and Cafiso, 1986; Lu et al., 1995). Due to the disordered chain ends, the entire surface at which both layers meet appears very rough, much rougher than the side surfaces of the acyl chains close to their headgroups. Therefore, the lipid mobility is mainly determined by interlayer friction and much less by intralayer viscosity (Horner et al., 2013).

In summary, recent experimental and theoretical findings suggest that interdigitation is not required to interleaflet-couple lipid nanodomains. On the other hand, close contacts and frequent interactions of acyl chains belonging to opposing leaflets seem to play a more relevant role. The degree of mixing across the bilayer midplane thus depends on the length of both *sn*-1 and *sn*-2 acyl chains, the presence of double bonds, and acyl chain asymmetry (Capponi et al., 2016). Although these interactions across the midplane appear significant, their importance still needs to be confirmed by detailed MD simulations, and possibly by experimental work.

### Cholesterol

In the past, it has been often suggested that cholesterol flip-flop, i.e., the rapid exchange of cholesterol between the two leaflets, can significantly contribute to domain registration (Hamilton, 2003). The proposed mechanism would result primarily from the fact that cholesterol can move significantly faster in the disordered phase in comparison to the ordered one (Risselada and Marrink, 2008). Therefore, higher cholesterol flip-flop rates are monitored if less/more ordered domains in one leaflet are matched with less/more ordered domains in the other leaflet. In contrast, cholesterol movement will be confined to only one leaflet in case of nonmatching domains. However, and although the contribution of cholesterol flip-flop to the overall coupling energy might seem considerable, by far it does not reach the contributions reported for the remaining coupling mechanisms (see the sub-sections above) (May, 2009). Thus, cholesterol flip-flop can be presently excluded as a plausible mechanism for nanodomain registration.

Yet, according to a recent coarse grain simulation study by Thallmair et al. (2018), cholesterol might still be involved in lipid domain registration, but in a slightly different manner. This study identified an intermediate state of cholesterol, in which it is sandwiched between the leaflets. This state would be responsible for increased correlations in lipid densities between the two leaflets, resulting in a weak repulsion of L_*o*_ domains and a small attraction of the remaining liquid disordered phase, thereby promoting domain registration (panel C in Figure 1). Although this finding agrees well with another simulation study (Weiner and Feigenson, 2018), it still requires experimental confirmation.

## Experimental Evidence for the Registration of Micro- and Nanodomains

Microdomains have immense practical advantage over much smaller nanodomains, mostly thanks to their adequate size for conventional fluorescence microscopy. More sophisticated approaches had to be developed to enable lipid domain detection and characterization at the nanoscale (Eggeling et al., 2009; Heberle et al., 2016; Šachl et al., 2016; Koukalová et al., 2017). Nevertheless, studying the registration of these nanodomains is even more challenging, since high resolution is, in principal, required not in two but in all three directions simultaneously. The choice of approach to be used for this purpose is thus very limited. In this section, we summarize the most important contributions of the techniques that, from our standpoint, contributed the most to our understanding of interleaflet coupling in model membranes.

### Imaging

**FIGURE 3:**
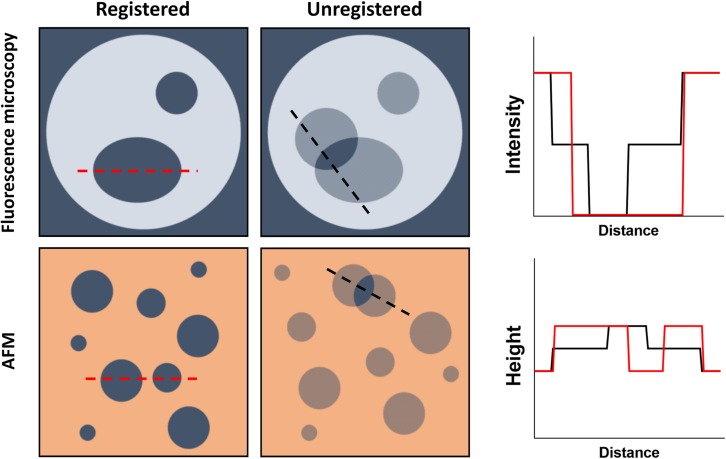
Schematic representation of the direct visualization of lipid domains by fluorescence microscopy and AFM. **(Top)** Domain formation within GUVs can be assessed by fluorescence microscopy if, for example, a lipid dye with no affinity for the domains is used. For registered domains, the bulk membrane fluorescence is recorded while the domains have no intensity. Unregistered domains would present half the fluorescence intensity of the bulk membrane whenever there is no corresponding domain within the opposing leaflet. **(Bottom)** Using AFM, L_*o*_ nanodomains, for example, can be detected by measuring the height of the bilayer, since they will be thicker than the bulk membrane. Unregistered domains would however present an intermediate membrane height resulting from the opposing bulk membrane not contributing to the thickness increase.

Direct visualization of lipid domain registration can be accomplished by AFM-based approaches or several different variations of fluorescence microscopy. It requires, however, the ability to detect a third intermediate state that might arise from having a domain in one leaflet without a counterpart in the opposing layer. In practice, this is accomplished by measuring the height of an SPB (thickness) with AFM or the fluorescence intensity arising from both leaflets in a fluorescence microscopy experiment (Figure 3).

Due to its high 3D resolution, AFM has been the most used imaging tool to study interleaflet coupling. The general coupling of both lipid monolayers has been studied by tracking the temperature-induced phase transition of the bilayer (thickness). Several studies reported two transition temperatures instead of one, meaning that both leaflets would respond independently (uncoupled) to variations in temperature (Keller et al., 2005; Seeger et al., 2009). However, it is now evident that interleaflet coupling in supported bilayers greatly depends on the SPB preparation conditions and the strength of the interaction between the solid support and the proximal leaflet (type of and distance from the support). It was shown, for example, that by increasing the preparation temperature of 1-palmitoyl-2-oleoyl-*sn*-glycero-3-phosphoethanolamine (POPE)/1-palmitoyl-2-oleoyl-*sn*-glycero-3-phospho-(1′-rac-glycerol) (POPG) SPBs (assembled on mica by vesicle fusion), it is possible to couple both monolayers and thus obtain a single-phase transition for the bilayer, accompanied by the formation of registered domains (Seeger et al., 2009). Interestingly, assembling the same bilayer onto a silicon oxide solid support always resulted in a single temperature-induced phase transition independently of the preparation procedure, pointing to coupling of both leaflets (Seeger et al., 2010).

It is then expected that studying micro- and nanodomain interleaflet coupling in SPBs will also be affected by the same additional contingencies. Indeed, Lin et al. (2006) have shown that using three distinct preparation procedures to form 1,2-dilauroyl-*sn*-glycero-3-phosphocholine (DLPC)/1,2-distearoyl-*sn*-glycero-3-phosphocholine (DSPC) supported bilayers resulted in different patterns of DSPC-enriched gel domain registration: all registered (1.8 nm above the DLPC surroundings), all unregistered (1.2 nm above), and registered/unregistered (1.8 and 1.1 nm above, respectively). Apart from the preparation protocol, the distance (*d*) at which the bilayers are assembled onto the support also seems to greatly interfere with the degree of domain registration, as clearly demonstrated by Garg et al. (2007). In their work, 1-stearoyl-2-oleoyl-*sn*-glycero-3-phosphocholine (SOPC):eggSph:Chol (1:1:1) SPBs were prepared using a solid support (*d* = 15 Å) or a hydrophilic polymer cushion at *d* = 30 Å or *d* = 58 Å. Using epifluorescence microscopy, the authors show that complete registration of L_*o*_ domains across the bilayer could only be achieved when the bilayer was sufficiently decoupled from the solid support, in this case at 58 Å (Garg et al., 2007).

Although a systematic study of interleaflet coupling at different lipid compositions is still missing, several imaging studies have over the years retrieved pertinent information on particular aspects of domain registration. For example, DOPC:Sph supported bilayers contain domains that extend 1 nm over the bulk fluid membrane (Rinia et al., 2001). However, intermediate heights are also detected, suggesting that domains in both monolayers are independent from each other and thus uncoupled. Interestingly, when cholesterol is included in the bilayer [DOPC:Sph (1:1) + 25 mol% Chol], these intermediate height levels are no longer detected and the domains appear 0.8 nm above the overall membrane, indicating that cholesterol is involved in L_*o*_ domain registration (Rinia et al., 2001). Since cholesterol flip-flop is already excluded as a possible mechanism for cholesterol-induced interleaflet coupling (May, 2009), these results tend to support coarse grain simulations showing that cholesterol might be sandwiched between both leaflets and by that promote domain registration (see section “Cholesterol”) (Thallmair et al., 2018; Weiner and Feigenson, 2018).

Nevertheless, moving from SPBs to free-standing bilayer models, and thus avoiding the effect of the support, seems to result in more consistent data. In 1,2-diphytanoyl-*sn*-glycero-3-phosphocholine (DiphyPC)/1,2-dipalmitoyl-*sn*-glycero-3-phosphocholine (DPPC)/Chol vesicles (different compositions along a tie-line), as well as cell-derived giant plasma membrane vesicles (GPMVs), lipid domains appear always in-register along the bilayer normal (Cornell et al., 2018). In practice, no intermediate fluorescence intensity levels were observed, confirming the interleaflet coupling of the domains both in giant unilamellar vesicles (GUVs) and GPMVs (Cornell et al., 2018). Similar results were obtained in GUVs composed of DLPC/DPPC/Chol (Korlach et al., 1999). Furthermore, when directly compared, vesicles and SPBs present a very different behavior. Contrary to what is observed in GUVs, phase-separated SPBs composed of DOPC:DPPC:Chol and DOPC:brainSph:Chol are static and do not couple across the bilayer over an experimental timescale of 2–3 h (Stottrup et al., 2004).

Overall, most fluorescence microscopy and AFM data show that micro- and nanodomains in both leaflets are coupled across the bilayer both in free-standing vesicles and in SPBs where the support does not play a significant role.

### Shear Stress Experiments

The aim of a shear stress experiment is to measure the shear that is required to move microdomains in an SPB out of registry (Blosser et al., 2015). The shear is applied by a hydrodynamic flow above the lipid bilayer. A 10 μm large domain in an SPB that is 1 nm apart from the solid surface is exposed to an effective friction that is approximately 1,000 times larger than the interleaflet friction (Bayerl and Bloom, 1990; Kiessling and Tamm, 2003). Consequently, the collective motion of the lipids in the lower leaflet is inhibited and remains hindered even when the shear is applied.

Shear stress experiments show that the shear required to deregister domains increases with the decrease in domain size (Blosser et al., 2015). This suggests that nanodomains (<200 nm diameter) must be registered. However, since the experiments were carried out in a relatively narrow range of domain sizes (1.5–6 μm), interleaflet coupling of lipid domains at the nanoscale is solely inferred by extrapolating these results across a wider size range. Nevertheless, this consideration is fairly useful as it allows at least a partial characterization of the nanodomains, which is generally difficult due to their small size.

### Förster Resonance Energy Transfer Analyzed by Monte Carlo Simulations

**FIGURE 4:**
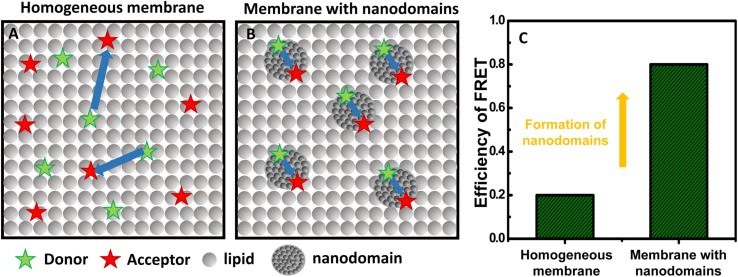
Basic principles of MC-FRET for detection of nanodomains. **(A)** At a sufficiently high acceptor concentration in the bilayer, FRET between donors (green stars) and acceptors (red stars) occurs. **(B)** If donors and acceptors with high affinity for nanodomains are used, the presence of nanodomains leads to accumulation of donors and acceptors in the nanodomains, and consequently to a more efficient FRET. **(C)** The efficiency of FRET on a homogeneous vs a heterogeneous membrane with nanodomains.

**FIGURE 5:**
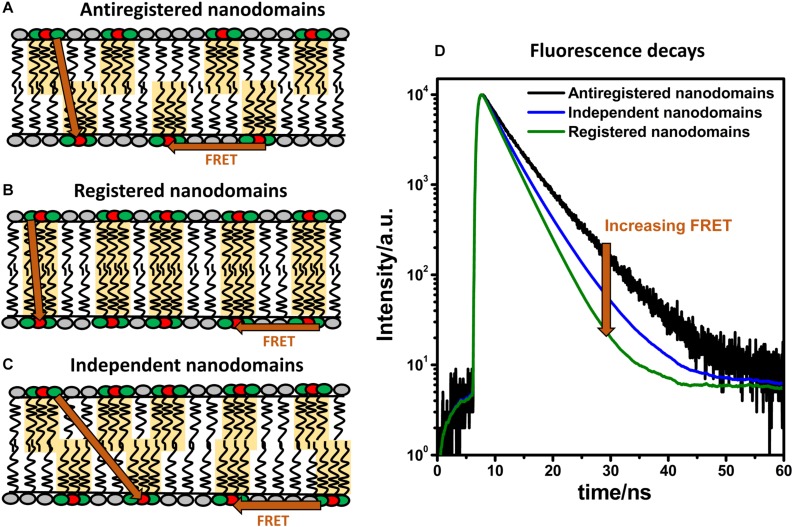
FRET and domain registration. In a lipid bilayer, FRET (indicated by orange arrows) occurs within the same leaflet (intra-FRET) but also from one leaflet to the other one (inter-FRET). The efficiency of FRET, measured by time-resolved spectroscopy, depends on mutual organization of nanodomains. The efficiency of FRET is the lowest when nanodomains are in antiregistration **(A)**, intermediate when they are independent **(B)**, and the highest when the nanodomains are registered **(C)**. Time-resolved fluorescence decays of donors in the presence of acceptors shown on panel **(D)** report on the kinetics of deexcitation and contain information about the size and concentration of nanodomains and their interleaflet organization.

As described above, studying interleaflet coupling of nanodomains requires high spatial resolution to allow the distinction of registered and unregistered domains within the membrane. Recently, we have shown that MC-FRET fulfills such requirements (Vinklárek et al., 2019). In the past, we have mainly used the method to determine the size of nanodomains and the total area occupied by these lipid domains (Šachl et al., 2015; Amaro et al., 2016; Koukalová et al., 2017). However, when an appropriate donor/acceptor pair is used, MC-FRET can also resolve interleaflet coupled from independent nanodomains. More specifically, MC-FRET relies on the use of fluorescently labeled lipids (which act as either donors or acceptors for energy transfer) with high affinity for the nanodomains (Figure 4) (Šachl et al., 2011, 2012). Thus, in the presence of these nanodomains, both donors and acceptors accumulate locally, leading to an efficient FRET process. Importantly, if the donor/acceptor pair is selected so that its Förster radius matches the thickness of the lipid bilayer, FRET will occur not only within the same leaflet but also across the membrane, from one leaflet to the other (Figure 5) (Vinklárek et al., 2019). These FRET events along the membrane normal make it then possible to study interleaflet coupling of nanodomains, since their frequency depends on the spatial interleaflet organization of the domains.

As shown on Figure 5, the outcome of an MC-FRET measurement is the time-resolved fluorescence decay of donors recorded in the presence of acceptors (Valeur, 2001). As a rule of thumb, the average lifetime of the donors is shorter and the decay faster the more often FRET occurs. Importantly, the decay becomes significantly faster when nanodomains are formed, with the extent to which this happens depending on their interleaflet arrangement: the highest when nanodomains are registered and the lowest in case of anti-registration (Figure 5). The shape of the decay thus contains information not only about the nanodomain size (expressed in terms of nanodomain radius *R*_*D*_) and the total area occupied by the nanodomains but also on their interleaflet coupling.

Using this method, we were able to show that nanodomains found in DOPC/Chol/SM bilayers containing 10 mol% of oxidized phospholipids are registered for a broad range of nanodomain sizes ranging from 10 to 160 nm (Vinklárek et al., 2019). The possibilities that the nanodomains in each monolayer were independent of each other, anti-registered or in partial registration were clearly excluded. Therefore, this work represents the first experimental evidence that nanodomains, like microdomains, are registered in free-standing bilayers for a broad spectrum of nanodomain sizes. Additionally, it is worth noting that MC-FRET is not limited to symmetric bilayers. Studies are underway which could demonstrate that MC-FRET can be used to study formation of nanodomains individually in each leaflet.

### Diffusion Techniques

Lipid domain coupling across the membrane implies that any hinderance in lipid diffusion (due to nanodomain formation) in one leaflet must be accompanied by an equivalent effect in the opposing layer. In principle, diffusion techniques such as FCS or single-particle tracking (SPT) can be used to retrieve reliable information on the extent of domain registration if both leaflets can be measured independently. This can be achieved by either preparing asymmetric bilayers with distinct labeled lipids in each layer or by obstructing lipid diffusion in one side of the bilayer and tracking how it translates to the opposing leaflet. A good example is the use of FCS to measure lipid diffusion on asymmetric vesicles with the inner leaflet composed of PC lipids [labeled with 1,2-dioleoyl-*sn*-glycero-3-phosphoethanolamine (7-nitro-2-1,3-benzoxadiazol-4-yl), NBD-DOPE] and the outer leaflet comprising Sph (labeled with Atto647-Sph) (Chiantia and London, 2012). In this study, the authors tested DOPC, OMPC, POPC, and SOPC (increasing T_*m*_) in the inner layer and brain Sph (shorter chain and weak interdigitation), milk Sph (longer chain and strong interdigitation), and synthetic C24:0 Sph on the outer leaflet. The presence of Sph decreases the diffusion within the outer leaflet and can then be used to study if and how this decrease translates to a slower lateral diffusion within the inner leaflet, i.e., to understand if the leaflets are coupled (see section “Chain Interdigitation” for details on interdigitation). While the above-mentioned FCS study required the formation of bilayers which are selectively labeled either in the inner or outer layer, fluorescence lifetime correlation spectroscopy (FLCS) (Kapusta et al., 2007) allows to simultaneously monitor the diffusion in both layers labeled with the same fluorescently labeled lipid analog. The main requirement for such FLCS experiment is to create a difference in the fluorescence lifetime of the dye when located in one of the two lipid layers. One possibility is to use potassium iodide to reduce the fluorescence lifetime in one layer, which might in turn change the diffusion properties of that layer (Vácha et al., 2009; Otosu and Yamaguchi, 2019). Metal induced energy transfer (MIET) represents a more universal approach. Here, the lipid bilayers are adsorbed onto an indiumtinoxide-covered glass (Benda et al., 2006; Przybylo et al., 2006). More recently, graphene has also been used as the “metal” layer yielding distinct lifetime differences between both layers (Ghosh et al., 2019). Although no systematic experiments on interleaflet coupling were performed so far, we believe that the combination of FLCS with MIET will be a useful tool to quantify interleaflet coupling.

Apart from using asymmetric bilayers, binding of proteins and polymers has been used to hinder lipid diffusion in one leaflet and study interleaflet coupling. For example, binding of poly-L-lysine (PLL) to one leaflet of a planar lipid bilayer was shown by FCS to decrease lipid diffusion and to attract a PLL-bound slow-diffusing patch on the opposing leaflet, resulting in nanodomain registration (Horner et al., 2009). In a different approach using wide-field single molecule fluorescence imaging, pinning of an SOPC bilayer through polymer-tethered phospholipids, and thus hindering the diffusion of the proximal leaflet, also resulted in a slower diffusion of the lipopolymer-free leaflet (Deverall et al., 2008). Overall, nanodomain interleaflet coupling, as seen by different diffusion techniques, appears to result from the balance between the freedom of the membrane to undulate and the energy required to maintain the domains in-register across the bilayer.

### Small-Angle Neutron Scattering

Together with the approaches described above, neutron scattering has been extensively used to study lipid domain formation at the nanoscale (Czeslik et al., 1997; Nicolini et al., 2004; Pencer et al., 2005, 2006, 2007a,b; Hirai et al., 2006; Masui et al., 2006, 2008; Krivanek et al., 2008; Vogtt et al., 2010; Heberle et al., 2013). Since neutrons are mainly scattered by their interaction with atomic nuclei (Pabst et al., 2010), the scattering process has very different sensitivities for isotopes of the same element. Thus, an obvious advantage over fluorescence techniques is the minimal perturbation that results from not using probes to address, for example, membrane lateral organization. In biomolecular studies, such as lipid domain formation, hydrogen and deuterium are the most used isotopes, with coherent neutron scattering lengths of −3.742 and −6.674 fm, respectively (Marquardt et al., 2015). In general terms, this scattering contrast forms the basis of any small-angle neutron scattering (SANS) experiment and can be optimized by either varying the hydrogen/deuterium ratio of the buffer and/or by selectively deuterating specific parts of a particular component of the studied system (Pan et al., 2013). In membrane organization studies, both the headgroup and the acyl chains can be labeled.

Nevertheless, studying nanodomain formation with SANS greatly depends on matching the scattering from the solvent and the lipid vesicles and on enhancing the contrast between the domain and nondomain phases. Otherwise, the contrast between the bulk solution and the vesicles would overwhelm any signal arising from lipid domain formation (Pan et al., 2013). Therefore, the main goal of a typical SANS experiment is to obtain a strong scattering signal arising from the contrast between the bulk membrane and the lipid nanodomains. This is frequently achieved by deuterating a specific lipid species with high affinity for the nanodomains, e.g., the acyl chain of the lipid with the highest melting temperature. In this way, the scattering signal would depend only on the size, composition, and shape of the nanodomains. For comprehensive reviews on the use of SANS to detect nanodomain formation (see Pan et al., 2013; Marquardt et al., 2015).

Recently, SANS has also been shown to be suitable for addressing coupling of nanodomains across the bilayer (Nickels et al., 2015a; Heberle et al., 2016; Heberle and Pabst, 2017; Eicher et al., 2018). The experimental strategy often used is based on rendering one of the phases invisible to neutrons by contrast matching (either the nanodomains or the bulk membrane) and on comparing the bilayer thicknesses and bending moduli of both homogeneous and heterogeneous vesicles. Using this strategy, Nickels et al. (2015a) were able to demonstrate that L_*d*_ nanodomains (∼13 nm) within POPC/DSPC/Chol large unilamellar vesicles (LUVs; 60 nm) were in-register across the bilayer. By contrast matching the L_*o*_ phase of the phase-separated vesicles, the resulting scattering signal allowed the determination of the thickness and the bending modulus of only L_*d*_ nanodomains. The obtained values were identical to the ones obtained for the bulk L_*d*_ phase of the homogeneous vesicles, confirming interleaflet coupling and the concomitant registration of the nanodomains. Since the L_*o*_ phase was not contributing to the scattering wave in this case, domain antiregistration would alternatively result in bilayer thickness values corresponding to a monolayer (only the L_*d*_ leaflet would be detected) and higher bending modulus of the L_*d*_ nanodomains, since they would be across the L_*o*_ phase on the opposing leaflet. Similar strategies have also shown, e.g., that disordered inner leaflets can fluidize ordered nanodomains within the outer layer (Heberle et al., 2016) and that intrinsic lipid curvature (e.g., POPE) can play a role in coupling both leaflets (Eicher et al., 2018). Since both these studies use asymmetric bilayers, they are discussed in more detail in section “Domain Registration in Asymmetric Bilayers.”

### Molecular Dynamic Simulations

Although computer sciences have recently experienced unprecedented development, computational power is still very limited for applications related to nanodomain coupling. Most importantly, the recently available computational power does not allow (1) to include enough lipid molecules into an atomistic simulation to simulate multiple domains at the same time; and (2) to simulate domain registration for a sufficiently long time. Despite these shortcomings, MD simulations represent an important complementary approach in domain studies, because they can, apart from the above-mentioned continuum theories (see section “Membrane Undulations”), provide a detailed molecular view.

To better demonstrate the limitations of MD simulations, let us assume a simulation box that contains a bilayer in which each leaflet contains five nanodomains with 10 nm radius occupying in total 50% of the bilayer surface. Under these conditions, the entire box contains approximately 8,700 lipid molecules, out of which 440 molecules belong to just one nanodomain. Furthermore, a molecule needs approximately 11 μs to fully cross a nanodomain and 90 μs to reach the other end of the box. The possibilities of an atomistic simulation are clearly determined by considering a typical simulation which runs for around 3 μs and contains about 512 lipids (Enkavi et al., 2019).

Scientists usually overcome this limitation by using coarse grain simulations, which inevitably lead to less accurate results. Together with the difficulties to account for macroscopic phenomena (such as membrane undulations) in the simulations, this may explain why nanodomain antiregistration has mainly been observed by *in silico* approaches (Stottrup et al., 2004; Crane et al., 2005; Stevens, 2005; Lin et al., 2006; Bennun et al., 2007; Perlmutter and Sachs, 2011; Williamson and Olmsted, 2015a, b). Specifically, Perlmutter and Sachs (2011) studied registration of nanodomains by coarse grain simulations in various mixtures of saturated (di-16:0 or di-20:0) and unsaturated (di-18:2) lipids with cholesterol. They observed almost perfect antiregistration in the mixtures that contained the same amounts of L_*o*_ and L_*d*_ phases. As shown on Figure 2, perfect antiregistration is only possible in such mixtures, leading to the release of stress enforced onto the membrane by hydrophobic mismatch. If the L_*o*_ content is higher or lower than the L_*d*_, it inevitably leads at least to partial phase overlap or to a better mixing of the saturated and unsaturated lipids, resulting in the suppression of phase separation (Perlmutter and Sachs, 2011). Interestingly, when the authors increased the content of L_*o*_ phase, they observed some regions with L_*o*_/L_*o*_ overlap but also increased mixing of saturated with unsaturated lipids at domain boundaries. On the other hand, when the content of L_*d*_ was higher than the content of L_*o*_, frequent registration of L_*d*_ regions was detected. In a different work, long-term domain antiregistration has also been observed by Stevens, who reported transbilayer matching of lipids with short tails with those possessing long tails (Stevens, 2005). In other cases, antiregistered nanodomains were found only as metastable structures during nucleation of nanodomains and the consequent formation of the final equilibrium state comprising registered nanodomains (Williamson and Olmsted, 2015a, b).

In contrast to the above mentioned work based on coarse grain simulations, Javanainen et al. (2017) performed atomistic MD simulations on an experimentally well characterized binary system of DPPC and cholesterol. The simulation was 1.3 μs long and contained 1,000 lipids. A phase diagram for this system is available and predicts the coexistence of L_*o*_ and L_*d*_ phases at approximately 10 to 20 mol% of cholesterol and temperatures slightly above the transition temperature of DPPC. Importantly, the authors observed the formation of nanodomains with 5 nm radius and a strong correlation in lipid chain ordering indicative of domain registration. Similarly, registered nanodomains were observed in a coarse grain simulation of a DPPC/DOPC/Chol (64/16/20) bilayer (Thallmair et al., 2018). In this work, cholesterol sandwiched between the leaflets was suggested to contribute to the registration of nanodomains. In addition, using both coarse-grained and all-atom MD simulations of membranes composed of DPPC, cholesterol and 1,2-diundecanoyl-*sn*-glycero-phosphocholine (DUPC; including variations on the position of the double bonds), Zhang and Lin (2019) have also shown L_*o*_ domain registration depending on the position of the acyl chain *cis* double bonds. Given the great diversity of simulation results, it seems that more atomistic simulations will need to be carried out, especially for the membranes that are according to coarse grain simulations prone to the formation of antiregistered nanodomains.

## Domain Registration in Asymmetric Bilayers

Despite the existence of protocols for preparation of asymmetric vesicles, studies focusing on interleaflet coupling of nanodomains in asymmetric bilayers are rare. Interesting work has been carried out on the extent to which the properties of one leaflet are influenced by the different properties of the opposing leaflet. Kiessling et al. (2006) showed more than a decade ago that L_*o*_ microdomains in one layer induce phase-separation in the opposing layer composed of porcine brain PC, PE, PS, and cholesterol. However, the same domains are not enough to form domains in the opposite leaflet consisting solely of POPC and cholesterol. Similarly, Collins and Keller (2008) demonstrated that asymmetric bilayers composed of varying amounts of DiphyPC, DPPC, and cholesterol, in which only one leaflet has a composition that drives phase-separation, can either be microscopically phase-separated, or homogeneous, depending on the ratio between the three lipids. Recently, Heberle et al. (2016) performed SANS experiments on asymmetric vesicles where the outer leaflet was composed of DPPC and the inner leaflet of POPC. The authors managed to show that the order of the outer leaflet gel domains was significantly decreased by coupling to the inner leaflet POPC. In contrast, the DPPC gel domains did not alter the packing density of the POPC inner leaflet. Remarkably, experiments performed by Wang and London (2018) indicate that increasing the ability of the outer leaflet to form L_*o*_ phase by itself (e.g., increasing the high-T_*m*_ PC content) decreases the inhibition of L_*o*_ domain formation in the outer leaflet by inner leaflet lipids.

Based on these experiments, it seems that both the chemical composition and temperature are important parameters that govern interleaflet coupling. Wan et al. (2008) showed that interleaflet coupling strongly depends on intrinsic chain melting temperatures (thereby on the chemical composition and temperature) and to a much lesser extent on the specific headgroup classes. Interestingly, it follows from a recent work by Eicher et al. (2018) that interleaflet coupling is also mediated by bilayer curvature. In this work, the authors prepared asymmetric LUVs where the inner and outer leaflets were composed of POPE and POPC, respectively. Strong coupling was demonstrated by cooperative melting and similar packing of both inner and outer leaflets. In contrast, when the composition of both leaflets was reversed, i.e., the inner leaflet contained POPC and the outer one POPE, the melting transition became broad and the coupling disappeared. The authors interpreted their results by a less convenient arrangement of POPE in the latter case, which – despite its conical shape was forced to reside in the outer layer. These data thus provide evidence for curvature-mediated interleaflet coupling in asymmetric bilayers.

Overall, the above mentioned experimental results suggest that some degree of interleaflet coupling indeed exists but is not very strong. If both layers showed no coupling or were only weakly coupled, they would have to be completely independent of each other. Nearly all the above experimental results speak against this conclusion. On the other hand, very strong coupling would predict that – irrespective of bilayer composition – both layers would need to behave as one single unit with a sharp melting temperature roughly corresponding to an intermediate value for both leaflets. This is evidently not the case. Therefore, the level of interleaflet coupling must be intermediate. This is concomitant with the observed characteristic behavior which includes: considerable sensitivity of coupling to external parameters such as temperature, and a strength of coupling that is dependent on acyl chain composition, packing of individual leaflets, bilayer curvature and the overall chemical composition of the lipid bilayer.

## Implications for Cellular Membranes

**FIGURE 6:**
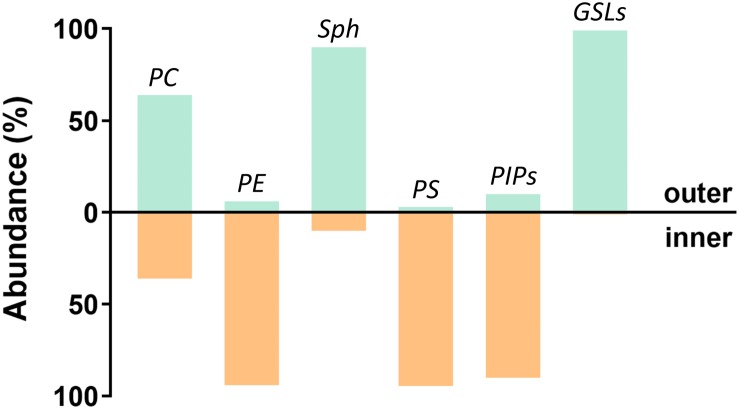
Plasma membrane asymmetry. Schematic representation of the plasma membrane lipid composition. Distribution in both leaflets is depicted as a percentage of the total lipid class. Based on (Zachowski, 1993; Lingwood, 2011; Fujimoto and Parmryd, 2017).

Cell membranes are much more complex when compared to model membranes (Figure 6). They contain not only a larger amount of different lipids, but also proteins that can have a significant influence on the resulting strength of interleaflet coupling. Nevertheless, the experiments performed on model systems show that coupling is strong enough, even in the absence of proteins, to induce domain registration in a wide range of domain sizes. Except for a few MD simulations and experimental AFM studies (Rinia et al., 2001; Stevens, 2005; Lin et al., 2006; Garg et al., 2007; Perlmutter and Sachs, 2011), micro- as well as nanodomains in symmetric bilayers have been mostly found in registration. Such behavior obviously requires an effective mechanism that can register domains irrespective of their size. So far, two mechanisms have been identified as being the most efficient: membrane undulations and line tension, which complement each other (see sections “Line Tension” and “Membrane Undulations” for details). On the other hand, the importance of acyl chain interdigitation as an efficient coupling mechanism has not yet been fully established, as discussed in section “Chain Interdigitation.”

It can be expected that in cellular membranes the differences in lipid packing and order between nanodomains and the remaining bulk membrane will be less pronounced than in model membranes. Nevertheless, we have recently reported that nanodomains with subtle differences to their surroundings are also formed in model systems and that these nanodomains are interleaflet coupled (Koukalová et al., 2017). This implies that large differences between the physical properties of the nanodomain and non-domain parts are generally not required, and that domain registration could be a part of biological membranes as well. Since cell membranes also oscillate and contain areas with increased line tension, the mechanism by which such in-register domains are formed in cellular membranes can hypothetically be based on similar principles, although other mechanisms are also very likely to contribute.

It should not be overlooked that biological membranes are asymmetric, contrasting with the most used model systems (Figure 6). Synthetic asymmetric membranes emerged as a completely new type of membrane mimetics about 10 years ago and have since become very popular. Experiments performed in recent years have shown that interleaflet coupling is strong, but not always strong enough that domains formed in one layer would induce formation of domains in the other layer of generally different composition. Cases have been reported where the presence of lipid domains in one layer resulted in different effects within the opposing layer: (1) formation of domains (Garg et al., 2007; Collins and Keller, 2008; Kiessling et al., 2009), (2) formation of areas of reduced lipid mobility (Chiantia and London, 2012), or (3) no change detected (Eicher et al., 2018). These results indicate that individual leaflets interact, but the final equilibrium state depends on temperature, acyl chain composition, packing of individual leaflets, bilayer curvature, and the overall chemical composition.

The conditions where a small change in one or more physical parameters can modulate the strength of coupling seem ideal for lipid domain registration to play a non-negligible role within cell membranes. For instance, it has been suggested that interleaflet coupling could be used to transmit signals from the outer to the inner membrane leaflet. This mechanism has often been discussed in connection with signaling pathways initiated by the binding of protein ligands to the outer layer (Klokk et al., 2016; Skotland and Sandvig, 2019). Probably the best-known examples are the binding of Cholera toxin to ganglioside GM_1_ (Wernick et al., 2010), Shiga toxin to globotriaosylceramide Gb_3_ (Johannes and Römer, 2010; Bergan et al., 2012), and the binding of lectins to GSL (Wang et al., 2009; Russo et al., 2016; Ledeen et al., 2018). Moreover, the virus SV40 is known to multivalently bind GM_1_ (Ewers et al., 2010). In the case of Cholera toxin, it has been shown experimentally that its binding to GM_1_ induces formation of L_*o*_ nanodomains (Štefl et al., 2012). The presence of such regions with impeded mobility of lipids and proteins is sufficient to dynamically segregate proteins into these regions (Nicolau et al., 2006). The existence of such regions, with possible registration across the bilayer, could therefore facilitate nanoscale protein–protein interactions and be important for many signaling cascades.

Nevertheless, the mechanism through which lipid nanodomain formation in one leaflet is then transferred to the opposing lipid layer ultimately leading to signal transduction across the membrane in living cells is still not yet clear. More specifically, both peripheral and transmembrane proteins, due to their abundance and function, are bound to play a role in the overall process. One example is the influence the actin cytoskeleton seems to have in the organization of outer plasma membrane lipids and GPI-anchored proteins. Although the experimental evidence is still sparse, lipid pinning of inner plasma membrane lipids (and other proteins) by the cytoskeleton has been suggested to be involved in the transduction process. In general, pinning accounts for the reduced or absent mobility of membrane components, which in turn alters the mixing entropy of the membrane and could lead to phase separation. MD simulations have shown that immobile molecules can act as obstacles to the diffusion of the remaining mobile membrane components, ultimately resulting in the formation of in register nanodomains (Fischer et al., 2012). At the plasma membrane of living cells, the actin cytoskeleton has been shown to interact directly with negatively charged lipids such as phosphatidylinositol 4,5-bisphosphate (PIP_2_), reducing their mobility and inducing the formation of GM_1_-containing L_*o*_ nanodomains in the outer leaflet (Dinic et al., 2013; Fujimoto and Parmryd, 2017). This explains why, in plasma membrane blebs lacking actin cytoskeleton filaments, the fraction of these GM_1_-containing L_*o*_ domains significantly decreases (Dinic et al., 2013). It is worth noting that pinning, or reduced lipid mobility due to protein binding, also occurs within the outer plasma membrane leaflet. As previously stated, the most studied case is the cross-linking of GM_1_ and the consequent formation of L_*o*_ patches frequently including GPI-anchored proteins – the so-called rafts (Dinic et al., 2013; Fujimoto and Parmryd, 2017). Interestingly, some studies seem to indicate that upon cross-linking (pinning), GM_1_ acyl chains might stretch and increase the contacts with inner leaflet lipids at the bilayer midplane (Spillane et al., 2014; Sun et al., 2015). This, as discussed in section “Chain Interdigitation,” would definitely favor registration of nanodomains formed through, e.g., binding of cholera toxin to the outer leaflet GM_1_ molecules. The importance of membrane midplane interactions became even more evident in a very comprehensive study by Raghupathy et al. (2016). Using a combination of FCS, fluorescence recovery after photobleaching (FRAP), and anisotropy measurements in CHO cells, as well as lipidomics and atomistic MD simulations, the authors show that not only long saturated acyl chains are required to patch GPI-anchored proteins but also PS molecules with long acyl chains at the inner plasma membrane leaflet are required for domain interleaflet coupling. Moreover, MD simulations showed that immobilizing long saturated acyl chains, irrespectively of the leaflet, stabilizes cholesterol-dependent transbilayer interactions within patches of the membrane with biophysical properties characteristic of a L_*o*_ phase (Raghupathy et al., 2016). This work is in great alignment with the results obtained in model systems, reinforcing the role of membrane midplane interactions in nanodomain registration (see sections “Chain Interdigitation” and “Cholesterol”). Apart from protein–lipid interactions, also cell adhesion to biological or non-biological surfaces has been shown to induce lipid nanodomain registration (Gordon et al., 2008). In this case, interaction with the surface leads to an increased stiffness and decreased fluctuations at the contact regions. In other words, cell adhesion could promote lipid demixing and domain formation in the outer membrane leaflet. As discussed in sections “Line Tension” and “Membrane Undulations,” a local increase in line tension and decreased undulations could then induce the formation of nanodomains in the opposing leaflet, even if the lipid composition is not prone to phase-separate (Gordon et al., 2008).

In addition to peripheral proteins, also transmembrane proteins have been suggested to help forming and stabilizing lipid nanodomains across the membrane, thus being intimately involved in interleaflet coupling and signal transduction. Recently, using a mean-filed lattice-based model, Bossa et al. (2019) suggest that the effect of introducing a transmembrane domain (TMD) within the membrane very much depends on the strength of the lipid–protein interactions. The authors show that, if the interactions are weak, lipids get diluted by the TMD and no domains are formed. If, on the other hand, the interaction strength is in the range of lipid–lipid interactions (responsible for domain formation), then the TMD has the ability to couple the domains across the bilayer (Bossa et al., 2019). Nevertheless, the most common effect of incorporating a TMD within a membrane is the possible hydrophobic mismatch and the consequent increase in line tension. However, since transmembrane proteins have frequently very important and conserved functions (such as membrane receptor), cells have evolved to decrease this energetic penalty, by properly modulating the orientation of the TMDs. For example, negatively charged lipids (e.g., PS and PIP_2_) of the inner plasma membrane leaflet associate mainly with positive regions of TMDs, in order to accommodate the local protein charge and hydrophobic thickness (von Heijne and Gavel, 1988; Gafvelin et al., 1997; Lin and London, 2014; Nickels et al., 2015b). Besides charge, also the thickness of TMDs seems to be adjusted to membrane asymmetry and involved in lipid domain coupling. It has been suggested that thin TMDs (rich in Ala/Gly) might be more easily incorporated into ordered and tightly packed domains than thicker TMDs rich in Leu/Phe. Indeed, Lorent et al. (2019) have recently shown that plasma membrane TMDs are in general asymmetric. Specifically, to avoid great perturbations of the outer, tightly packed, plasma membrane leaflet, exoplasmic portions of TMDs tend to be thinner. This observation suggests that, by keeping the asymmetry, TMDs could, in principle, couple ordered lipid nanodomains on the exoplasmic leaflet (e.g., containing GM_1_) with more fluid patches at the cytoplasmic leaflet (e.g., containing PIP_2_), thus assuring direct and efficient signal transduction. In addition, recent MD simulations also show that by migrating to lipid domain boundaries, transmembrane proteins are able to reduce the line tension in 25–35%, once again suggesting the energetic balance resulting from having a TMD within the membrane might indeed stabilize the lipid domains and their registration (Bandara et al., 2019).

In conclusion, it should be noted that this review focused mainly on domain interleaflet coupling induced by lipids in protein-free bilayers. It is clear, however, that particular membrane proteins can play a significant role in nanodomain coupling. Specially, transmembrane proteins can act as transducers of lipid assembly, by not only influencing interleaflet coupling and nanodomain registration but also by modulating the size of these domains (Yethiraj and Weisshaar, 2007), attracting some lipids/proteins (Anderson and Jacobson, 2002; Marsh, 2008) or influencing mechanistic properties of lipid bilayers that are responsible for nanodomain coupling (see section “Theoretical Framework: Mechanisms Leading to the Nanodomain Registration”). Despite a clear significance of proteins for lipid domain interleaflet coupling, the literature on this topic is very limited. On the other hand, significant attention was paid to the formation of the so-called lipid shells around proteins (see Marsh, 2008 for a comprehensive summary). These shells can be characterized as regions with reduced lipid mobility and specific lipid order and composition. Nevertheless, and despite its biological importance, this field is relatively unexplored and should deserve more attention in the near future.

Overall, the purpose of this review was to provide the reader with a comprehensive biophysical view of the problematics of interleaflet coupling. So far, biophysical studies show that interleaflet coupling is strong enough to drive registration of lipid domains of any physically relevant size and that the strength of coupling depends on the specific conditions in which the membrane is currently found. Based on this knowledge, it seems likely that interleaflet coupling can play a significant role in cell membrane lateral organization and processes related to signal transduction across the membrane.

## Author Contributions

MS and RŠ conceived the idea and wrote the manuscript with the help of MH.

## Conflict of Interest

The authors declare that the research was conducted in the absence of any commercial or financial relationships that could be construed as a potential conflict of interest.

## Abbreviations

AFM: atomic force microscopy
CHO cells: Chinese hamster ovary cells
Chol: cholesterol
DiphyPC: 1,2-diphytanoyl-*sn*-glycero-3-phosphocholine
DLPC: 1,2-dilauroyl-*sn*-glycero-3-phosphocholine
DOPC: 1,2-dioleoyl-*sn*-glycero-3-phosphocholine
DPPC: 1,2-dipalmitoyl-*sn*-glycero-3-phosphocholine
DSPC: 1,2-distearoyl-*sn*-glycero-3-phosphocholine
DUPC: 1,2-diundecanoyl-*sn*-glycero-phosphocholine
FCS: fluorescence correlation spectroscopy
FLCS: fluorescence lifetime correlation spectroscopy
FRAP: fluorescence recovery after photobleaching
FRET: Förster resonance energy transfer
GPI: glycosylphosphatidylinositol
GPMV: giant plasma membrane vesicle
GSL: glycosphingolipids
GUV: giant unilamellar vesicle
L_*d*_: liquid-disordered
L_*o*_: liquid-ordered
LUV: large unilamellar vesicle
MC-FRET: Förster resonance energy transfer analyzed by Monte Carlo simulations
MD: molecular dynamic
MIET: metal induced energy transfer
NBD-DOPE: 1,2-dioleoyl-*sn*-glycero-3-phosphoethanolamine (7-nitro-2-1,3-benzoxadiazol-4-yl)
OMPC: 1-oleoyl-2-myristoyl-*sn*-glycero-3-phosphocholine
PC: phosphatidylcholine
PE: phosphatidylethanolamine
PIP: phosphatidylinositol phosphate
PIP_2_: phosphatidylinositol 4,5-bisphosphate
PLL: poly-L-lysine
POPC: 1-palmitoyl-2-oleoyl-*sn*-glycero-3-phosphocholine
POPE: 1-palmitoyl-2-oleoyl-*sn*-glycero-3-phosphoethanolamine
POPG: 1-palmitoyl-2-oleoyl-*sn*-glycero-3-phospho-(1′-rac-glycerol)
PS: phosphatidylserine
SANS: small-angle neutron scattering
SPB: supported phospholipid bilayer
Sph: sphingomyelin
SOPC: 1-stearoyl-2-oleoyl-*sn*-glycero-3-phosphocholine
T_*m*_: melting temperature
TMD: transmembrane domain.
